# Supplementary material for: Hyperspectral anomaly detection leveraging spatial attention and right-shifted spectral energy
Source: PLoS One. 2025 Sep 4;20(9):e0330640. doi: 10.1371/journal.pone.0330640 (PMC12410889; doi:10.1371/journal.pone.0330640)
Supplement: Attachment — Submitted filename: Comments on PONE-D-25-02392.pdf [file pone.0330640.s001.pdf]

In this manuscript, the authors propose a hyperspectral anomaly detection method that integrates spatial and spectral information by combining graph attention network with a Beta wavelet graph neural network. Below are some suggestions aimed at further enhancing the quality and readability of the manuscript:

1. **Originality of Figures**: In the section titled "Right-Shift Phenomenon", Figure 1 appears highly similar to a figure in the published article "Hyperspectral Anomaly Detection Based on a Beta Wavelet Graph Neural Network" (IEEE Multimedia, vol. 31, no. 2, pp. 69–79, Apr.–Jun. 2024). To ensure the originality of the manuscript, it is recommended that the authors make necessary modifications to Figure 1 or provide a more detailed explanation to distinguish it from the existing work.
2. **Algorithm Pseudocode**: Although the authors have provided a thorough description of the algorithm, to facilitate readers' quick understanding of the algorithm's workflow, it is suggested that a complete and clear pseudocode flowchart with standardized notation be added. This would significantly enhance the readability and reproducibility of the manuscript.
3. **Label Acquisition**: Based on the description of Equation (17), the proposed method appears to be a supervised method. However, obtaining labels for hyperspectral anomaly detection is typically challenging. The authors are encouraged to elaborate on how these labels were acquired, specifically whether the ground truth maps of the data were used for training. This would help readers better understand the feasibility and practicality of the method.
4. **Ablation Experiments**: The manuscript lacks comparative experiments on the effects of the KNN parameter *k* and the performance without the graph attention network. It is recommended that the authors supplement relevant ablation experiments to more comprehensively evaluate the performance of the proposed method.
5. **Computational Complexity Analysis**: In the "Computational Complexity Analysis" section, the authors have provided a detailed analysis of the computational complexity of various algorithms. However, directly presenting the actual runtime of each algorithm on the dataset would offer a more intuitive understanding of the performance of the proposed method.
6. **Spelling Error Correction**: The authors are advised to carefully check the spelling throughout the manuscript. Specifically, on page 2, line 35 of the Introduction section, there is a noticeable spelling error ("Itt"), which should be corrected.
7. Some new works highly related to this topic are suggested to be referred, such as:
   HADGSM: A unified nonconvex framework for hyperspectral anomaly detection. IEEE Transactions on Geoscience and Remote Sensing, 2024, 62, 5503415.
   BS^3LNet: A new blind-spot self-supervised learning network for hyperspectral anomaly detection. IEEE Transactions on Geoscience and Remote Sensing, 2023, 61, 5504218.
   Hyperspectral anomaly detection based on chessboard topology. IEEE Transactions on Geoscience and Remote Sensing, 2023, 61, 5505016.
   Information entropy estimation based on point-set topology for hyperspectral anomaly detection. IEEE Transactions on Geoscience and Remote Sensing, 2024, 62, 5523415.
