# Supplementary material for: Hyperspectral anomaly detection leveraging spatial attention and right-shifted spectral energy
Source: PLoS One. 2025 Sep 4;20(9):e0330640. doi: 10.1371/journal.pone.0330640 (PMC12410889; doi:10.1371/journal.pone.0330640)
Supplement: Attachment — Submitted filename: Comments to PONE-D-25-02392.pdf [file pone.0330640.s002.pdf]

Comments to PONE-D-25-02392

General Comment:

This paper proposes a novel method that integrates a Graph Attention Network with a Beta wavelet neural network, offering a new perspective on hyperspectral anomaly detection. The proposed method demonstrates promising results across multiple datasets and in comparison with several benchmark methods. However, there are certain shortcomings that need to be addressed. Therefore, major revisions are recommended. The detailed comments below outline specific areas for improvement.

Detail comments:

1. The literature review lacks recent studies on hyperspectral anomaly detection. The latest references only extend to 2023, with no citations from 2024. It is recommended to include more up-to-date studies.
2. The main contributions should be more concise. It is suggested to condense them into three key points.
3. The structure of the MLP and AGG functions in the network propagation process (Equations 12–16) is not clearly specified. Details such as the number of layers and activation functions in the MLP, as well as whether AGG performs concatenation or summation, should be provided.
4. There is a lack of discussion on the rationale behind parameter selection. The algorithm section should include a justification for the choice of

the k value in KNN. Additionally, the specific parameter settings for the Beta wavelet (e.g., values of a and b) and their impact on the results should be elaborated.

5. Many of the contrastive learning methods cited in the paper are outdated. It is recommended to incorporate more recent methods from the past three years to better highlight the novelty and advantages of the proposed approach.
6. The evaluation metrics are too limited, as they only include AUC and ROC comparisons. Table 1, as well as Figures 3, 5, 7, 9, 11, 13, 15, and 17(a), all present the same AUC comparison, which increases the length of the paper without adding significant information. It is suggested to include 2D (PD,τ), 2D (PF, τ), and 3D-ROC curve comparisons to better illustrate the relationship between the detection rate and false alarm rate.
7. It is recommended to add boxplots to better visualize the separation between background and anomalies.
8. The paper claims that the Beta wavelet avoids Laplacian matrix decomposition (e.g., in Equation 9), yet the equations still involve U (the eigenvectors of the Laplacian matrix). The authors should clarify how the decomposition step is bypassed.
9. The paper lacks a comparison of inference times. It is recommended to include this aspect for a more comprehensive evaluation.

10. The limitations of the proposed algorithm are not sufficiently discussed. For instance, on the AVIRIS-I dataset, the algorithm performs worse than CRD. The authors should explain the possible reasons for this and suggest potential improvements.
