# Supplementary material for: Hyperspectral anomaly detection leveraging spatial attention and right-shifted spectral energy
Source: PLoS One. 2025 Sep 4;20(9):e0330640. doi: 10.1371/journal.pone.0330640 (PMC12410889; doi:10.1371/journal.pone.0330640)
Supplement: Attachment — Submitted filename: Comments to PONE-D-25-02392R1-v1.pdf [file pone.0330640.s004.pdf]

# Comments to PONE-D-25-02392R1

1. The manuscript exhibits inconsistent and non-standard use of abbreviations. Terms such as hyperspectral anomaly detection (HAD) and graph attention network (GAN) are repeatedly redefined throughout the paper, which is not acceptable in academic writing. Abbreviations should be introduced only once—upon first use—and used consistently thereafter. The authors are encouraged to revise the manuscript thoroughly to ensure proper and standardized use of abbreviations.
2. The literature review section in the Introduction is overly detailed and lacks conciseness. It is recommended that the authors streamline this section by emphasizing the key techniques, their limitations, and how the proposed method addresses existing gaps, rather than providing lengthy explanations of each related work.
3. On page 15, line 416, the manuscript refers to ". Figure ??", which suggests a citation or rendering error. All figure references should be correctly formatted and verified.
4. Figure 8 appears to be incomplete or missing content in the current version of the manuscript.
5. The phrase "the Proposed method" appears multiple times with incorrect capitalization. Please revise all instances to "the proposed method".
6. In the first paragraph of the Computational Complexity Analysis section, there are several occurrences of inconsistent or excessive spacing between words.
