# Supplementary material for: Hyperspectral anomaly detection leveraging spatial attention and right-shifted spectral energy
Source: PLoS One. 2025 Sep 4;20(9):e0330640. doi: 10.1371/journal.pone.0330640 (PMC12410889; doi:10.1371/journal.pone.0330640)
Supplement: Attachment — Submitted filename: Response to Reviewers.docx [file pone.0330640.s003.docx]

Dear Reviewers,

Thank you for your thorough review and insightful comments on our manuscript. We have carefully considered each point and made the necessary revisions to improve the quality of our work.To facilitate your review, we have highlighted all the modified sections in yellow within the manuscript. We appreciate your time and effort in evaluating our paper and hope that our revisions meet your expectations.

Sincerely, Ruhan A

**reviewer 1**

This paper proposes a novel method that integrates a Graph Attention

Network with a Beta wavelet neural network, offering a new perspective

on hyperspectral anomaly detection. The proposed method demonstrates

promising results across multiple datasets and in comparison with several

benchmark methods. However, there are certain shortcomings that need to be addressed. Therefore, major revisions are recommended. The detailed comments below outline specific areas for improvement.

Detail comments:

1. The literature review lacks recent studies on hyperspectral anomaly

detection. The latest references only extend to 2023, with no citations

from 2024. It is recommended to include more up-to-date studies.

Respond：Thank you for your suggestion. We have added several recent studies from 2024 and 2025 to ensure the literature review is comprehensive and up-to-date.

2. The main contributions should be more concise. It is suggested to

condense them into three key points.

Respond：We sincerely appreciate your suggestion to streamline the main contributions of our work. In response, we have condensed them into three key points to enhance clarity and focus. Thank you for your valuable feedback.

3. The structure of the MLP and AGG functions in the network

propagation process (Equations 12–16) is not clearly specified. Details

such as the number of layers and activation functions in the MLP, as

well as whether AGG performs concatenation or summation, should be

provided.

Respond：Thank you for your valuable feedback regarding the structure of the MLP and AGG functions in our network propagation process. We sincerely appreciate your suggestions and have addressed them by providing detailed specifications in the "Experiments" section.

4. There is a lack of discussion on the rationale behind parameter selection. The algorithm section should include a justification for the choice of the k value in KNN. Additionally, the specific parameter settings for the Beta wavelet (e.g., values of a and b) and their impact on the results should be elaborated.

Respond：Thank you for your valuable feedback. Following your suggestions, we have added a "Parameter Analysis" section to the paper, discussing the selection of k values in KNN and the settings of Beta wavelet parameters (such as a and b) and their impact on the results. We hope these additions meet your expectations.

5. Many of the contrastive learning methods cited in the paper are

outdated. It is recommended to incorporate more recent methods from

the past three years to better highlight the novelty and advantages of

the proposed approach.

Respond：We sincerely appreciate your insightful comments regarding the cited contrastive methods. To address your suggestion, we have incorporated more recent methods into our comparisons, including PDBSNet (2023), BockNet (2023), DirectNet (2024), and NL2Net (2025), into our comparative analysis. Thank you again for your valuable feedback.

6. The evaluation metrics are too limited, as they only include AUC and

ROC comparisons. Table 1, as well as Figures 3, 5, 7, 9, 11, 13, 15, and

17(a), all present the same AUC comparison, which increases the

length of the paper without adding significant information. It is

suggested to include 2D (PD,τ), 2D (PF, τ), and 3D-ROC curve

comparisons to better illustrate the relationship between the detection

rate and false alarm rate.

Respond：We sincerely appreciate your feedback regarding the evaluation metrics. To address your suggestion, we have removed the redundant figures (3, 5, 7, 9, 11, 13, 15, and 17(a)) and added comparisons using 2D (PD,τ), 2D (PF, τ), and 3D-ROC curves. These new metrics are now presented in Figures 2, 5, 8, 11, 14, 17, and 20 to better illustrate the relationship between detection rate and false alarm rate. Thank you again for your valuable comments.

7. It is recommended to add boxplots to better visualize the separation

between background and anomalies.

Respond：We sincerely appreciate your suggestion to enhance the visualization of the separation between background and anomalies. In response, we have added boxplots to Figures 4, 7, 10, 13, 16, 19, and 22. Thank you for your valuable feedback.

8. The paper claims that the Beta wavelet avoids Laplacian matrix

decomposition (e.g., in Equation 9), yet the equations still involve U

(the eigenvectors of the Laplacian matrix). The authors should clarify

how the decomposition step is bypassed.

Respond：Thank you very much for your valuable comments and suggestions regarding our paper. You raised an important point about the Beta Wavelet avoiding the decomposition of the Laplacian matrix while the equations still involve $U$(the eigenvectors of the Laplacian matrix). We are more than happy to provide further clarification.

In theory, the Beta Wavelet transform is indeed expressed as $W_{a,b}=U\beta_{a,b}^{*}\left( \Lambda\right)U^{T}$, where$U$represents the eigenvectors of the Laplacian matrix L. However, in practice, explicit computation of U is not required. The key insight is that the Beta Wavelet kernel $\beta_{a,b}^{*}\left( L \right)$ is designed as a polynomial function of the Laplacian matrix L, which allows us to perform computations directly in the spatial domain without the need for explicit eigendecomposition. More specifically, the Beta Wavelet kernel is formulated as:

$$\beta_{a,b}^{*}\left( L \right)=\frac{\left( L/2 \right)^{(a-1)}\left( I-L/2 \right)^{(b-1)}}{2B\left( a,b \right)}$$

By recursively computing the powers of L (e.g.,$\left( L/2 \right)^{(a-1)}$ and $\left( I-L/2 \right)^{(b-1)}$), we can efficiently implement the polynomial kernel, thus avoiding the high computational cost associated with explicit eigendecomposition.

We understand that the presence of U in the equations might cause confusion, as it is included for theoretical completeness. To address this, we have revised the methodology section of the paper to provide a more detailed explanation of how the Beta Wavelet transform is achieved through polynomial approximations without explicitly computing the eigenvectors matrix U.

Once again, we sincerely appreciate your insightful feedback and are committed to improving our paper based on your suggestions.

9. The paper lacks a comparison of inference times. It is recommended to

include this aspect for a more comprehensive evaluation.

Respond：We sincerely appreciate your suggestion regarding the comparison of inference times. In response, we have included this aspect in the "Computational Complexity Analysis" section. Thank you for your valuable feedback.

10. The limitations of the proposed algorithm are not sufficiently discussed. For instance, on the AVIRIS-I dataset, the algorithm performs worse than CRD. The authors should explain the possible reasons for this and suggest potential improvements.

Respond：We sincerely appreciate your feedback regarding the limitations of our proposed algorithm. In response, we have added a discussion on the possible reasons for the algorithm's suboptimal performance on the AVIRIS-I dataset. Thank you for your valuable comments.

**reviewer 2**

In this manuscript, the authors propose a hyperspectral anomaly detection method that integrates spatial and spectral information by combining graph attention network with a Beta wavelet graph neural network. Below are some suggestions aimed at further enhancing the quality and readability of the manuscript:

1. Originality of Figures: In the section titled "Right-Shift Phenomenon", Figure 1 appears highly similar to a figure in the published article "Hyperspectral Anomaly Detection Based on a Beta Wavelet Graph Neural Network" (IEEE Multimedia, vol. 31, no. 2, pp. 69–79, Apr.–Jun. 2024). To ensure the originality of the manuscript, it is recommended that the authors make necessary modifications to Figure 1 or provide a more detailed explanation to distinguish it from the existing work.

Respond：Thank you for your careful review and valuable feedback on the manuscript's figure originality. The article “Hyperspectral Anomaly Detection Based on a Beta Wavelet Graph Neural Network” you mentioned is my previous work. Figure 1 was an example of the right - shift phenomenon. To ensure originality and avoid content repetition, I've removed Figure 1. Thanks again for your constructive criticism.

2. Algorithm Pseudocode: Although the authors have provided a thorough description of the algorithm, to facilitate readers' quick understanding of the algorithm's workflow, it is suggested that a complete and clear pseudocode flowchart with standardized notation be added. This would significantly enhance the readability and reproducibility of the manuscript.

Respond：Thank you for your insightful suggestion. I fully recognize the importance of pseudocode in enhancing the readability and reproducibility of the manuscript. In response to your feedback, I have added a complete and clear pseudocode flowchart with standardized notation. Once again, I appreciate your constructive criticism, which has been invaluable in improving the quality of our work.

3. Label Acquisition: Based on the description of Equation (17), the proposed method appears to be a supervised method. However, obtaining labels for hyperspectral anomaly detection is typically challenging. The authors are encouraged to elaborate on how these labels were acquired, specifically whether the ground truth maps of the data were used for training. This would help readers better understand the feasibility and practicality of the method.

Respond：Thank you for your valuable feedback. We acknowledge the challenges of label acquisition in hyperspectral anomaly detection. In this work, labels were derived from standard ground truth maps of publicly available hyperspectral datasets. For training, only 40% of the labeled data was utilized to avoid overfitting, with the remaining 60% acting as unlabeled samples for anomaly score refinement. We thank you for your suggestion and have added a dedicated section in the Experiments part to detail the data sources and partitioning.

4. Ablation Experiments: The manuscript lacks comparative experiments on the effects of the KNN parameter k and the performance without the graph attention network. It is recommended that the authors supplement relevant ablation experiments to more comprehensively evaluate the performance of the proposed method.

Respond：Thank you for your feedback. We have added a "Parameter Analysis" section discussing the k value in KNN and an "Ablation Study" section comparing the performance with and without the graph attention network. We hope these additions address your concerns.

5. Computational Complexity Analysis: In the "Computational Complexity Analysis" section, the authors have provided a detailed analysis of the computational complexity of various algorithms. However, directly presenting the actual runtime of each algorithm on the dataset would offer a more intuitive understanding of the performance of the proposed method.

Respond：We sincerely appreciate your suggestion regarding the comparison of inference times. In response, we have included this aspect in the "Computational Complexity Analysis" section. Thank you for your valuable feedback.

6. Spelling Error Correction: The authors are advised to carefully check the spelling throughout the manuscript. Specifically, on page 2, line 35 of the Introduction section, there is a noticeable spelling error ("Itt"), which should be corrected.

Respond： We sincerely apologize for the oversight and confirm that the spelling error on page 2, line 35 ("Itt") has been corrected to "It." Thank you for your vigilance.

7. Some new works highly related to this topic are suggested to be referred, such as:

HADGSM: A unified nonconvex framework for hyperspectral anomaly detection. IEEE Transactions on Geoscience and Remote Sensing, 2024, 62, 5503415.

BS3LNet: A new blind-spot self-supervised learning network for hyperspectral anomaly detection. IEEE Transactions on Geoscience and Remote Sensing, 2023, 61, 5504218.

Hyperspectral anomaly detection based on chessboard topology. IEEE Transactions on Geoscience and Remote Sensing, 2023, 61, 5505016.

Information entropy estimation based on point-set topology for hyperspectral anomaly detection. IEEE Transactions on Geoscience and Remote Sensing, 2024, 62, 5523415.

Respond：Thank you for bringing these recent works to our attention. We have incorporated these references into the revised manuscript to ensure a comprehensive review of the latest advancements in hyperspectral anomaly detection.
