# Supplementary material for: Hyperspectral anomaly detection leveraging spatial attention and right-shifted spectral energy
Source: PLoS One. 2025 Sep 4;20(9):e0330640. doi: 10.1371/journal.pone.0330640 (PMC12410889; doi:10.1371/journal.pone.0330640)
Supplement: Attachment — Submitted filename: ResponseLetter.docx [file pone.0330640.s005.docx]

Dear Editors and Reviewers,

We sincerely appreciate your valuable time and constructive comments on our manuscript. All suggestions have been systematically addressed in the revised manuscript. Responses to your specific comments are provided in blue text below, while modifications in the manuscript are highlighted in yellow for easy tracking. These revisions significantly enhance the clarity and rigor of our work.

Sincerely, Ruhan A

Reviewer 1: This paper proposes a novel hyperspectral anomaly detection (HAD) algorithm, GAN–BWGNN HAD, which integrates a Graph Attention Network (GAN) and Beta Wavelet Graph Neural Network (BWGNN) to model spatial-spectral relationships. By leveraging graph structures and beta wavelets, the method enhances anomaly detection precision while improving computational efficiency. The suggestions can be considered:

1. The abstract should conclude with key experimental results to highlight the performance of the proposed method.

****Response: Thank you for your valuable suggestion. We have revised the abstract to include the key experimental results, as recommended, to better highlight the performance of our method. We appreciate your insightful feedback.****

2. The introduction should begin with a brief overview of hyperspectral remote sensing and hyperspectral imaging to provide context for readers unfamiliar with the domain.

****Response:Thank you for your thoughtful suggestion. We have revised the introduction to include a brief overview of hyperspectral remote sensing and imaging, providing essential context for readers unfamiliar with the domain. Your feedback greatly improved the accessibility of our work.****

3. The introduction should conclude with a brief summary of the paper’s structure to guide readers.

****Response:Thank you for your valuable suggestion. We have revised the introduction to conclude with a brief summary of the paper's structure, as recommended, to guide readers through the subsequent sections. Your feedback greatly enhanced the clarity of our manuscript.****

4. The proposed method appears to rely on labeled training data in Equation (17).The manuscript should clarify how labeled data is obtained for training.

****Response:** Thank you for your valuable suggestion. We have explicitly clarified how labeled training data is obtained in the "Datasets" section as recommended. Your feedback significantly strengthened our methodology description.**

5. The experiment section lacks details about the Grand Isle dataset, including sensor specifications and data source information.

**Response:Thank you for your insightful suggestion. We have supplemented the experiment section with Grand Isle dataset details (including sensor specifications and data sourcing).**

6. References 1 and 27 are duplicated.
**Response:Thank you for identifying the duplication in References 1 and 27. We sincerely apologize for this oversight and have fully revised the reference list to correct the error. Your meticulous review is greatly appreciated.**

7. The introduction lacks discussion on recent hyperspectral anomaly detection approaches, particularly those based on convolutional neural network (Global Feature-Injected Blind-Spot Network for Hyperspectral Anomaly Detection), low-rank and sparse representation (Hyperspectral Simultaneous Anomaly Detection and Denoising: Insights From Integrative Perspective), and generative adversarial networks (Frequency-to-spectrum mapping GAN for semisupervised hyperspectral anomaly detection).

****Response:Thank you for highlighting this critical gap. We have supplemented the introduction with discussions of recent hyperspectral anomaly detection approaches, including convolutional neural network (Global Feature-Injected Blind-Spot Network for Hyperspectral Anomaly Detection), low-rank and sparse representation (Hyperspectral Simultaneous Anomaly Detection and Denoising: Insights From Integrative Perspective), and generative adversarial networks (Frequency-to-spectrum mapping GAN for semisupervised hyperspectral anomaly detection). Your highly pertinent suggestions significantly strengthened the survey's comprehensiveness.****

Reviewer 2:

1. The manuscript exhibits inconsistent and non-standard use of abbreviations. Terms such as hyperspectral anomaly detection (HAD) and graph attention network(GAN) are repeatedly redefined throughout the paper, which is not acceptable in academic writing. Abbreviations should be introduced only once—upon first use—and used consistently thereafter. The authors are encouraged to revise the manuscript thoroughly to ensure proper and standardized use of abbreviations.

****Response:We sincerely apologize for the oversight in abbreviation usage. A systematic review of all abbreviations has been conducted throughout the manuscript to ensure standardized definition upon first occurrence (e.g., HAD, GAN) and consistent application thereafter, aligning fully with academic writing standards.****

2. The literature review section in the Introduction is overly detailed and lacks conciseness. It is recommended that the authors streamline this section by emphasizing the key techniques, their limitations, and how the proposed method addresses existing gaps, rather than providing lengthy explanations of each related work.

****Response:Thank you for this valuable suggestion to enhance conciseness. We have thoroughly streamlined the literature review in the Introduction by focusing on core techniques, their limitations, and our method's contributions to address existing gaps, significantly improving the section's clarity.****

3.On page 15, line 416, the manuscript refers to ". Figure ??", which suggests a citation or rendering error. All figure references should be correctly formatted and verified.

****Response: We sincerely apologize for the oversight in line 416 and have corrected the figure reference. All figure citations and references have been validated for correct formatting.****

4.Figure 8 appears to be incomplete or missing content in the current version of the manuscript.

****Response: We sincerely apologize for the oversight in Figure 8. This figure has been fully revised to complete its missing content . Thank you for your meticulous review – we have systematically verified all figures to enhance manuscript clarity.****

5. The phrase “the Proposed method” appears multiple times with incorrect

capitalization. Please revise all instances to “the proposed method”.

****Response:Thank you for catching the capitalization inconsistency. We have systematically revised all instances of "the Proposed method" to lowercase ("the proposed method") throughout the manuscript to ensure grammatical precision.****

6. In the first paragraph of the Computational Complexity Analysis section, there are several occurrences of inconsistent or excessive spacing between words.

**Response:Thank you for highlighting the spacing inconsistencies in the Computational Complexity Analysis section. We have standardized all word spacing throughout this paragraph to align with journal formatting guidelines, ensuring uniform typography.**
